# Clinical assessment meets laboratory science: adapting OSCE methodology for authentic biosciences evaluation in the age of generative AI

**DOI:** 10.1042/ETLS20253021

**Published:** 2026-03-24

**Authors:** Ruchira Mann, Sabrina Tosi, David Tree

**Affiliations:** 1 Department of Biosciences, Brunel University of London, Uxbridge, UB8 3PH, U.K.

**Keywords:** academic integrity, authentic assessment, generative AI, laboratory assessment, OSCE

## Abstract

The proliferation of generative artificial intelligence (AI) tools has fundamentally challenged traditional written assessments across higher education, with particular implications for laboratory-based disciplines where written work may substitute for demonstration of practical competence, necessitating approaches that prioritise direct performance. This study presents the adaptation of objective structured clinical examination (OSCE) methodology from medical education to laboratory biosciences, demonstrating a practical framework for authentic assessment in the AI era. We describe and evaluate the transformation of a microscopy assessment in FHEQ Level 4 Biomedical Sciences from a traditional laboratory report to a 20-minute OSCE-style practical evaluation. The redesigned assessment maintained grade distributions while eliminating AI vulnerability through real-time performance demonstration and conversational examination. The implementation achieved close alignment between learning outcomes and assessment methods while providing inherent resistance to generative AI exploitation through direct performance requirements. Equity implications are complex and context-dependent, with potential barriers for students with communication differences alongside potential benefits for others, such as those with written communication difficulties, emphasising the importance of balanced assessment portfolios and appropriate reasonable adjustments. The cross-disciplinary adaptation demonstrates that OSCE methodology offers a scalable solution to AI-era assessment challenges, with performance-focused design maintaining academic integrity more effectively than restrictive policies while enhancing authenticity and equity outcomes.

## Introduction

### The generative artificial intelligence challenge to assessment integrity

The rapid adoption of generative artificial intelligence (AI) tools has created a crisis of confidence in traditional assessment methods across higher education. While universities have developed policies on generative AI use, the generic nature of institutional guidelines creates a significant gap between policy and practice, leaving educators and students in specific disciplines with insufficient guidance on acceptable AI integration versus academic misconduct in their academic contexts [1].

This disruption has called into question whether traditional assessment methods like written assignments and reports can securely measure student performance. In laboratory-based disciplines, where practical skills and scientific reasoning are fundamental learning outcomes, the potential for AI to complete data analysis and written assessments/reports undermines the validity of these methods as evaluative tools, unless conducted under supervised conditions [2].

### Traditional assessment in laboratory sciences

Laboratory-based education in the biosciences has historically relied on a combination of practical work and written assessment, with laboratory reports serving as a primary mechanism for evaluating student understanding and skill development [3]. These assessments address multiple learning outcomes simultaneously: they can evaluate technical skills such as physical laboratory techniques and data collection; assess understanding of experimental design, scientific procedures and protocols; and promote internalisation of concepts through hands-on application. The laboratory sessions and report assessments provide valuable employability skills development, as they mirror the ‘authentic’ tasks and competencies a graduate will employ in professional scientific practice [4,5].

However, written laboratory reports have significant vulnerabilities in the AI era [6]. Students can use generative AI to analyse data, interpret results and construct arguments, making it difficult to distinguish between genuine student understanding and AI-assisted performance, rendering the assessment effectively invalid [7].

### OSCE methodology: a proven framework for authentic assessment

The objective structured clinical examination (OSCE) methodology, first introduced in medical education by Harden and colleagues in 1975 as an alternative to traditional clinical examinations, offers a robust framework for authentic assessment that addresses challenges posed by generative AI [8,9]. OSCEs assess performance through structured activities where candidates demonstrate competencies directly rather than through written communication, making assessment inherently AI-resistant while maintaining authenticity and validity [10]. The format enables assessment of multiple competencies within a single examination, providing holistic evaluation particularly valuable in laboratory sciences where students must demonstrate diverse technical skills, safety protocols and analytical techniques. See Table 1 for a summary of OSCE Features.

**Table 1:** Adaptation of core OSCE design principles for biosciences laboratory assessment, illustrating how established OSCE features were translated into a structured practical competency framework

Traditional OSCE features	Biosciences laboratory assessment adaptations
**Circuit of timed stations** (5–15 minutes each)	**Laboratory technique station** with specific time allocations for different procedures (e.g., 5 minutes for microscope operation, 7 minutes for specimen identification, 3 minutes for methodological discussion)
**Standardised patients** (real or simulated)	**Standardised laboratory scenarios** using consistent specimens, equipment setups and experimental conditions
**One-to-one examiner assessment**	**Direct observation** of individual student performance by trained demonstrators/academics
**Structured marking schemes** with checklists	**Laboratory competency checklists** covering technical skills, safety protocols and data interpretation
**Clinical skills assessment** (history taking, physical examination)	**Laboratory skills assessment** (equipment operation, experimental design, data collection and analysis)
**Communication skills evaluation**	**Scientific communication** assessment through verbal explanation of procedures, results interpretation and peer discussion
**Real-time performance observation**	**Live demonstration** of laboratory techniques, problem-solving and analytical thinking
**Standardised instructions** for each station	**Consistent laboratory protocols** and clear task instructions at each station
**Multiple competency domains** assessed simultaneously	**Integrated assessment** of theoretical knowledge, practical skills, safety awareness and scientific reasoning
**Immediate feedback** opportunities	**Formative questioning to elicit reasoning** during practical procedures
**Simulated clinical environments**	**Authentic laboratory settings** using actual equipment and realistic experimental conditions
**Professional behaviour assessment**	**Laboratory professionalism** including safety compliance, teamwork and ethical conduct

The table summarises general design adaptations and is not limited to the specific microscopy assessment described in this study. Some features represent potential design elements rather than components used in every implementation.

The assessment methodology described here shares similarities with objective structured practical examinations (OSPEs), which were first conceptualised as an adaptation of OSCEs for assessing practical skills in non-clinical laboratory settings [11]. OSPEs have been implemented and validated for assessment of laboratory exercises in preclinical sciences, particularly physiology [12]. Since then, OSPEs have been adopted across various laboratory-based disciplines including pharmacy, anatomy and biochemistry, demonstrating the versatility of structured practical assessment beyond clinical contexts [11]. While OSPEs traditionally focus primarily on structured evaluation of discrete technical skills, the adapted framework described in this study integrates technical performance with conversational assessment of analytical reasoning and theoretical understanding. In this sense, the approach retains core OSCE principles of holistic competency demonstration, integrated reasoning and examiner-guided exploration, applied within a laboratory context, rather than a purely technical model. Although the approach shares features with OSPE formats, it is framed primarily within the OSCE tradition because OSCE methodology is more extensively developed and theorised in the assessment literature.

### Constructive alignment, assessment validity and authenticity

OSCE-style assessments achieve superior constructive alignment between assessment tasks and learning outcomes compared with traditional written reports by requiring direct demonstration rather than indirect, proxy measures [13,14]. This direct accountability promotes authentic understanding through real-time application, eliminating the gap between what is assessed and what is learned. Assessment authenticity is strengthened by requiring students to demonstrate practical skills in laboratory scenarios mirroring professional practice, while direct observation provides higher validity by evaluating actual competencies rather than written communication abilities [15–17].

### Study context

We present here a case study of the transformation of a microscopy assessment in a Framework for Higher Education Qualifications (FHEQ) Level 4 Biomedical Sciences programme at Brunel University of London. We describe the development from traditional written laboratory reports to an OSCE-style practical evaluation, analysing implementation outcomes and examining implications for assessment design in the AI era. This study presents an adapted OSCE-informed assessment framework implemented through a practical assessment intervention, providing a design-oriented approach to performance-based assessment in laboratory disciplines in the context of generative AI.

## Methods

### Assessment context

The microscopy assessment addresses four learning outcomes: demonstrating understanding of microscopy and associated techniques; analysing and interpreting data; integrating knowledge from different sources; and working safely in the laboratory to obtain data (Table 2). The assessment was 20 credits until the 2024–25 year when it became 15 credits. In the UK higher education system, 20 credits correspond to approximately 200 notional learning hours. The module also includes a multiple-choice examination component; however, this study focuses specifically on the practical microscopy assessment intervention.

**Table 2:** Microscopy assessment learning outcomes

Demonstrate an understanding of the use of microscopy and associated techniques. The ability to analyse and interpret data. The ability to integrate knowledge and information gained from different sources to address a specific question. Work safely in the laboratory to obtain data.

### Data collection

Student performance data were obtained from institutional records for four academic years (2021–22 through 2024–25). These records included final grades under both the previous assessment format (2021–22 and 2022–23) and the revised OSCE-style format (2023–24 and 2024–25). Preliminary comparison of adjacent cohorts showed comparable grade distributions with no systematic differences prior to aggregation, supporting the decision to combine years within each assessment format for analysis. Grades for both years within a format were combined as they are sufficiently similar to justify aggregation. For the new format, years were statistically similar; for the old format, a small but statistically significant year-to-year difference was detected, likely reflecting Covid-related disruption in 2021 – 22 , but sensitivity checks confirmed that pooling did not alter the overall conclusions.

No personally identifiable information was used; all data were anonymised prior to analysis. Grades were compiled into a single dataset using Microsoft Excel and a summary table was created to display the distribution of grades. At Brunel University of London, the analysis and descriptive presentation of aggregated, anonymised student data does not require separate ethical approval. Students give permission for their data to be used for educational analysis on registration to study.

### Statistical analysis

To determine whether grade distributions differed between assessment formats, a Chi-squared test of independence was performed. Grade categories (A, B, C, D, E, F and Other) were treated as nominal variables, and assessment format (old vs. new) as the grouping variable. The old format included data from academic years 2021–22 (*n* = 235) and 2022–23 (*n* = 194), while the new format included 2023–24 (*n* = 142) and 2024–25 (*n* = 138). Percentage distributions for each grade category were converted to counts using cohort sizes for each year, forming the contingency table. The Chi-square test of independence was performed using Microsoft Excel to compare grade distributions. Effect size (Cramer’s V) was calculated manually using the Chi-square statistic and sample size.

### Traditional assessment and limitations

The microscopy assessment underwent multiple iterations over a decade-long period, reflecting evolving pedagogical approaches and practical constraints. The original format comprised a 1,500-word laboratory report structured around a hypothetical clinical case study that integrated knowledge across multiple laboratory disciplines including anatomy, histology, cell biology and microbiology. This multidisciplinary approach necessitated distribution across at least four specialist markers, creating significant challenges in maintaining consistency and requiring extensive moderation. The transition to digital submission platforms enabled more strategic allocation of marking responsibilities according to disciplinary expertise, with each assessment receiving evaluation from multiple specialists and final grades calculated as weighted averages. Subsequent iterations introduced automated quiz components featuring complex tasks including specimen identification, image labelling and quantitative analysis requiring formula application. These modifications, combined with a retained essay component examining microscopy applications and limitations, reduced marking demands and enabled broader marker participation. However, the increasing sophistication of generative AI tools rendered these components vulnerable to academic misconduct, fundamentally compromising assessment validity and necessitating alternative evaluation strategies.

### Assessment intervention design and implementation

The intervention implemented an adapted OSCE-informed structured practical assessment combining OSPE style technical competency evaluation with integrated conversational assessment of analytical reasoning and theoretical understanding through direct performance. To support student preparation, drop-in sessions were organised prior to the assessment, enabling students to develop proficiency in microscope operation, interpret histological slides and seek clarification from academic staff. Students also participated in guided practical sessions where they performed bacterial Gram staining and prepared blood smears using equine blood, followed by haematoxylin and eosin (H&E) staining. All preparations were examined under light microscopy. Students were additionally introduced to commercially prepared histology slides representing diverse organs and tissues, complementing content from the anatomy, histology and physiology module.

Assessment delivery employs a standardised 20 minute individual session structure (15 minutes assessment, 5 minutes examiner deliberation and feedback rubric completion) conducted in laboratory environments equipped with six stations, enabling simultaneous assessment of multiple students across parallel stations while maintaining individual evaluation integrity.

The examination protocol follows a structured progression through three phases: technical competence demonstration (microscope operation and component nomenclature), practical application (specimen examination and identification across magnifications), and theoretical integration (laboratory practical methodology discussion). Examiners employ a conversational approach designed to encourage elaboration and demonstrate understanding depth through guided questioning.

### Assessment setup and logistics

The assessment was conducted in a structured laboratory environment using six parallel stations, each staffed by two markers. Students completed a standardised 15 minute individual assessment followed by a brief scoring interval, enabling consistent evaluation across multiple concurrent stations while maintaining assessment integrity. To accommodate the full cohort, assessments were scheduled over four half-day sessions (each lasting three hours), allowing all students to complete the process under standardised conditions in half a week. To minimise opportunities for information sharing between students assessed on different days, 25–30 equivalent specimen sets of comparable difficulty were used at random. Performance across assessment sessions did not indicate systematic differences associated with assessment timing, suggesting no advantage for later participants. Across sessions, the station model enabled efficient assessment of large cohorts while maintaining double-marking and standardisation. Standardised equipment, anonymised specimens and controlled station separation ensured uniform assessment conditions.

### Examiner training and marking protocol

A double-marking approach was used to support reliability, with trained assessors calibrated through joint review of rubric descriptors, discussion of exemplar responses and alignment of grading expectations prior to assessment delivery. Structured questioning protocols guided progression from technical recognition to applied interpretation, enabling assessment of reasoning rather than recall. Grades were determined after scores from both markers were weighted equally and combined after discussion. Grading used a stepped marking scheme with fixed percentage values at regular intervals within each grade band (A*: 91-98%; A: 71-88%; B: 61-68%; C: 51-58%; D: 41-48%; E: 31-38%; F: ≤ 25%), explaining the absence of intermediate percentage values in the distribution.

Performance was evaluated using a structured rubric (Table 3) assessing technical operation, specimen interpretation and applied laboratory reasoning across graded performance levels. Analytical reasoning and data interpretation were assessed through students’ verbal explanation of specimen characteristics, interpretation of observed structures across magnifications and application of knowledge from laboratory practicals, ensuring alignment with competencies previously evaluated through written report analysis. Safe laboratory practice was assessed through direct observation of appropriate microscope handling, specimen management and adherence to laboratory conduct expectations during the assessment. Each criterion was assessed across six performance levels from excellent (A*/A+/A/A-) to very unsatisfactory (F), with detailed descriptors for each level. Students received written rubric-based feedback following completion of the assessment in line with standard institutional assessment procedures. This framework aligns with Institute of Biomedical Science standards for closed-book assessment while supporting authentic competence demonstration [18].

**Table 3:** Assessment Rubric

Criterion	Excellent (A/A+/A/A-)*	Very good (B-/B/B+)	Good (C-/C/C+)	Acceptable (D-/D/D+)	Unsatisfactory (E-/E/E+)	Very unsatisfactory (F)
**Ability to operate a light microscope**	Excellent ability to handle and operate the microscope with confidence; able to operate fine and coarse focus; confident with all parts; shows experience	Very good ability to handle and operate with confidence; able to operate fine and coarse focus; confident with all parts; shows familiarity	Can handle and operate with some confidence; able to operate fine and coarse focus; able to recognise most parts; would benefit from more practice	Attempted to handle and operate; able to operate fine and coarse focus; struggles to recognise some parts; would benefit from more practice	Attempted to handle and operate but struggles to find fine and coarse focus; struggles to recognise most parts; needs a lot of practice	Never seen a microscope before; unable to recognise any part including fine and coarse focus; needs a lot of practice and guidance
**Ability to identify microscopy specimens**	Excellent ability to identify specimens and describe the sample at fine details and different magnifications	Very good ability to identify specimens and describe the sample at different magnifications	Ability to identify specimens with some encouragement and describe some aspects at different magnifications	Ability to identify specimens with substantial help and describe some aspects using at least one magnification	Attempted to identify specimens but failed, although with help; could not describe the sample in sufficient detail	Failed to identify specimens, although with help; could not describe the sample in any detail
**Understanding of laboratory practicals that used the light microscope**	Excellent understanding; gave excellent description of a particular example including fine details of techniques involved	Very good understanding; gave very good description of a particular example including some relevant details of techniques involved	Good understanding; gave good description of a particular example; evidence of attendance to relevant practical sessions	Some relevant recollection of attending some practical sessions reflected in an approximate description of a particular lab practical	Evidence of having attended laboratory practicals; however, did not pay enough attention to retain relevant information	No evidence of having attended any laboratory practicals

## Results

### Outcomes and performance analysis

**Figure 1:**
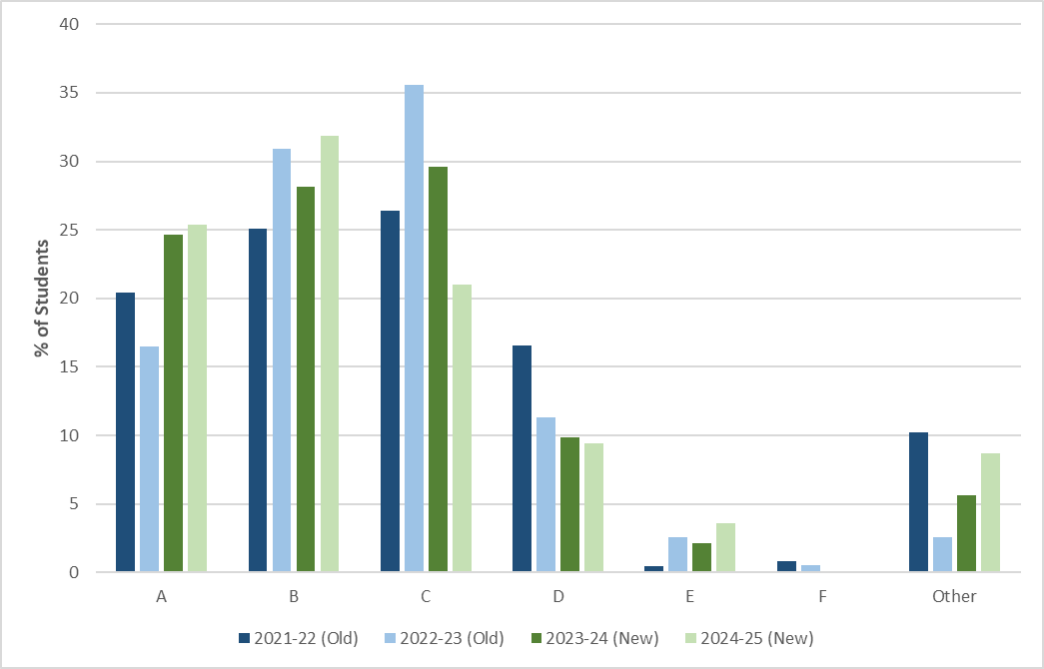
Shift in grade profiles with assessment redesign. Proportions of students achieving grades in the years before and after the introduction of the OSCE-style microscopy assessment. A grades are 71% and above, B grades 61%–68%, C grades 51%–58%, D grades 41%–48%, E grades 31%–38%, and F grades are 25% and below. The ‘Other’ category includes non-submissions and students on abeyance. Student cohort sizes are as follows: 2021–22 = 235 students, 2022–23 = 194 students, 2023–24 = 142 students and 2024–25 = 138 students.

Comparative analysis of grade distributions across the two years before and after transition to the in-person assessment reveals measurable changes in performance patterns following OSCE implementation (Figure 1). A Chi-square test of independence comparing grade distributions between the old format (2021–22 and 2022–23, *n* = 429) and the new format (2023–24 and 2024–25, *n* = 280) indicated no statistically significant difference in grade profiles (χ² = 11.81, df = 6, *P* = 0.066). However, the effect size, measured by Cramer’s V (0.13), suggests a small association between assessment format and grade distribution, indicating that overall academic standards were maintained while modest shifts in grade profile were consistent with direct assessment of practical competence rather than changes in grading standards. The successful transition occurred without substantial disruption to overall student outcomes, suggesting that the new assessment format maintained validity while achieving the intended pedagogical objectives of authentic performance demonstration.

Failure rates and non-attendance remained consistent across both assessment formats. While this suggests that the format change did not substantially alter overall student engagement at the cohort level, these aggregate measures cannot capture the nuanced ways in which different assessment formats may create or remove barriers for individual students with diverse needs (see Discussion). Prior to the new assessment format between five and ten students were referred each year for academic misconduct; there have been none after this change to in-person assessment.

## Discussion

### Implementation considerations and scalability

The assessment transition maintained alignment with intended learning outcomes while addressing contemporary challenges in assessment integrity through real-time demonstration of practical competence, reducing opportunities for inappropriate generative AI use. Conceptually, the approach occupies a hybrid position between OSCE and OSPE formats: drawing on the structured, station-based evaluation of technical competence characteristic of OSPEs, while extending this model through integrated reasoning, conversational probing and holistic competency demonstration aligned with OSCE methodology. As summarised in Table 1, this represents a structured adaptation of established design principles rather than a wholly new assessment taxonomy. Importantly, the intervention was not intended to replace written scientific communication, which remains essential and is developed elsewhere within the programme; instead, the OSCE-style format complements written assessment by enabling direct evaluation of practical and analytical competence.

Resource implications include increased direct contact time, especially as the assessment is double-marked, along with laboratory space requirements. The assessment was delivered using six stations with two markers per station (12 staff per session) across multiple sessions to accommodate cohorts of approximately 150–200 students. While the format requires concentrated staff involvement during delivery, the reduction in extended written marking and moderation resulted in overall workload comparable to previous written assessment formats.

Following reflection on the first year of implementation, several refinements were introduced to improve efficiency and assessor well-being. Scheduled breaks were incorporated to reduce fatigue, and assessors rotated partners to enhance randomisation and maintain engagement. Pairing academic staff with members of the technical team proved highly beneficial: technicians were able to pose detailed questions regarding practical sessions attended by students, enriching the assessment process and providing valuable additional perspectives on student laboratory performance. Future developments include training a larger pool of academic assessors to ensure continuity in the event of unforeseen absences and to accommodate anticipated increases in student enrolment. Additionally, pre-assessment discussions with the technical team are planned to optimise the configuration of practical stations and ensure consistency across sessions.

### OSCE assessment and equity challenges

As educators adapt assessment strategies in response to generative AI challenges, equity and inclusion must remain central considerations. Universities have legal obligations under the UK Equality Act 2010 to ensure assessments are equitable for all students. OSCE-style assessments introduce distinctive equity challenges, particularly for students with autism and communication differences who may find formats relying on rapid interpersonal exchanges and performance under time pressure disproportionately demanding [19,20]. This becomes problematic where intended learning outcomes focus on scientific reasoning rather than communication competence, creating potential misalignment between assessment procedure and learning objectives.

Reasonable adjustments were used to mitigate these issues: providing clear written instructions alongside verbal prompts, allowing extended station times or permitting alternative communication modes [21,22]. Examiner training is critical to ensure marking criteria focus on essential competences rather than neurotypical interaction norms [23].

### Equity benefits and assessment diversity

Conversely, OSCE-style assessments may address inequities faced by other disabled student groups. Dyslexic students often experience difficulties in written communication that mask underlying disciplinary knowledge and reasoning skills [24,25]. Students with executive function difficulties or mental health conditions may be disadvantaged by coursework deadline pressures [26]. Oral and practical assessments allow more direct competence demonstration without confounding factors from extended written communication or time management challenges.

The equity implications of OSCE-style assessment are interactional and context-dependent rather than uniformly beneficial. Assessment formats do not produce equity effects in isolation; instead, their impact emerges through the interaction between task design, learning outcomes and individual learner profiles. While time-pressured, performance-based assessment may present challenges for some students, including those with communication differences or heightened performance anxiety, it may reduce barriers for others by limiting reliance on extended written communication and independent time management. No single assessment mode can address the full range of learner variability, and all formats introduce distinct accessibility considerations. Consequently, equity in assessment is better understood as a property of balanced and intentionally designed assessment portfolios, supported by appropriate reasonable adjustments, rather than as an inherent feature of any individual assessment type. This interactional perspective aligns with Universal Design for Learning principles, which emphasise multiple means of action and expression as a mechanism for supporting diverse learners while maintaining academic standards [27,28].

### Competency-based learning outcomes and OSCE methodology

The implementation of OSCE-style assessment demonstrates strong alignment with competency-based education (CBE) frameworks, which emphasise demonstration of specific, measurable skills rather than written articulation [29,30]. The direct demonstration model enables authentic assessment of defined competencies while eliminating the confounding effects of written communication skills, as suggested in prior literature indicating that traditional written assessments may underestimate demonstrated practical competency [31].

### Constructive alignment and assessment validity

The grade distribution stability following implementation of OSCE-style assessment supports Biggs and Tang’s [13] constructive alignment theory by achieving better alignment between intended learning outcomes (practical laboratory competencies) and assessment methods (direct performance evaluation) compared with written proxies. Improved alignment between assessment tasks and intended competencies supports stronger assessment with clearer differentiation between theoretical knowledge and practical competence, demonstrating how improved alignment yields more accurate evaluation of student capabilities [17,32].

### Authentication and academic integrity in the digital age

Beyond addressing immediate generative AI concerns, the OSCE transformation represents a broader shift towards assessment design that is intrinsically resistant to AI-mediated substitution while preserving authentic demonstration of competence. The case study demonstrates that educators can maintain rigorous academic standards while eliminating opportunities for inappropriate AI assistance by refocusing on authentic performance demonstration, with stable grade distributions suggesting successful alignment between assessment methods and student competencies.

This approach offers a sustainable solution to the AI challenge that does not rely on detection technologies or honour systems, instead substantially reducing opportunities for misconduct through assessment design centred on real-time performance demonstration.

### Scalability and cross-disciplinary applications

This successful implementation suggests broader applicability across laboratory-based STEM disciplines, with principles like direct competency demonstration, conversational evaluation and structured practical tasks, adaptable in multiple subjects. While resource implications like direct contact time increased, the elimination of reading, marking time and academic misconduct investigations offset these costs.

### Future directions and policy implications

At Brunel, this approach is being applied to other assessments on the Biomedical Sciences BSc. Future work will examine optimal station design for different laboratory competencies and investigate long-term knowledge retention compared with traditional assessments [10,29]. For educational practice, this work demonstrates that technological disruption can catalyse pedagogical improvement rather than simply creating problems. The successful cross-disciplinary transfer from medical to laboratory education suggests that assessment design strategies focusing on authentic performance demonstration may be more effective than blanket AI restrictions for maintaining academic integrity [4,29].

## Conclusion

This study demonstrates that an adapted OSCE-informed assessment intervention can support authentic evaluation of laboratory competence while maintaining academic standards in the context of generative AI. The implementation preserved grade distributions, enabled direct demonstration of practical and analytical skills, and reduced vulnerability to AI-assisted completion. Importantly, the approach complements rather than replaces written assessment and highlights the value of diversified assessment portfolios. These findings suggest that performance-based, design-oriented assessment strategies offer a viable pathway for maintaining assessment validity and authenticity in laboratory education in the digital age. Rather than representing a wholly new assessment form, the approach demonstrates how established OSCE and OSPE principles can be productively integrated and adapted to contemporary laboratory education challenges.

## Open Access

This article has been published open access under our Subscribe to Open programme, made possible through the support of our subscribing institutions, learn more here: https://portlandpress.com/pages/open_access_options_and_prices#conditional

## Abbreviations

AI: artificial intelligence
CBE: competency-based education
OSCE: objective structured clinical examination
OSPEs: objective structured practical examinations
